# Effects of Mesenchymal Stromal Cell-Derived Extracellular Vesicles in Lung Diseases: Current Status and Future Perspectives

**DOI:** 10.1007/s12015-020-10085-8

**Published:** 2020-11-19

**Authors:** Haiyan Guo, Yue Su, Fang Deng

**Affiliations:** 1 Department of Pediatrics, The First Affiliated Hospital of Anhui Medical University, No. 218 Ji-Xi Road, 230022 Hefei, Anhui Province People’s Republic of China; 2 Wellcome-Wolfson Institute for Experimental Medicine, Queen’s University Belfast, 97 Lisburn Road, Belfast, Belfast, BT9 7BL UK; 3 Department of Nephrology, Anhui Provincial Children’s Hospital, Hefei City, Anhui Province 230022 People’s Republic of China

**Keywords:** Mesenchymal stromal cell, Extracellular vesicles, Clinical application

## Abstract

Mesenchymal stromal cells (MSCs) as a kind of pluripotent adult stem cell have shown great therapeutic potential in relation to many diseases in anti-inflammation and regeneration. The results of preclinical experiments and clinical trials have demonstrated that MSC-derived secretome possesses immunoregulatory and reparative abilities and that this secretome is capable of modulating innate and adaptive immunity and reprograming the metabolism of recipient cells via paracrine mechanisms. It has been recognized that MSC-derived secretome, including soluble proteins (cytokines, chemokines, growth factors, proteases), extracellular vesicles (EVs) and organelles, plays a key role in tissue repair and regeneration in bronchopulmonary dysplasia, acute respiratory distress syndrome (ARDS), bronchial asthma, chronic obstructive pulmonary disease (COPD), idiopathic pulmonary fibrosis (IPF), pulmonary arterial hypertension, and silicosis. This review summarizes the known functions of MSC-EV modulation in lung diseases, coupled with the future challenges of MSC-EVs as a new pharmaceutical agent. The identification of underlying mechanisms for MSC-EV might provide a new direction for MSC-centered treatment in lung diseases.

Graphical abstract
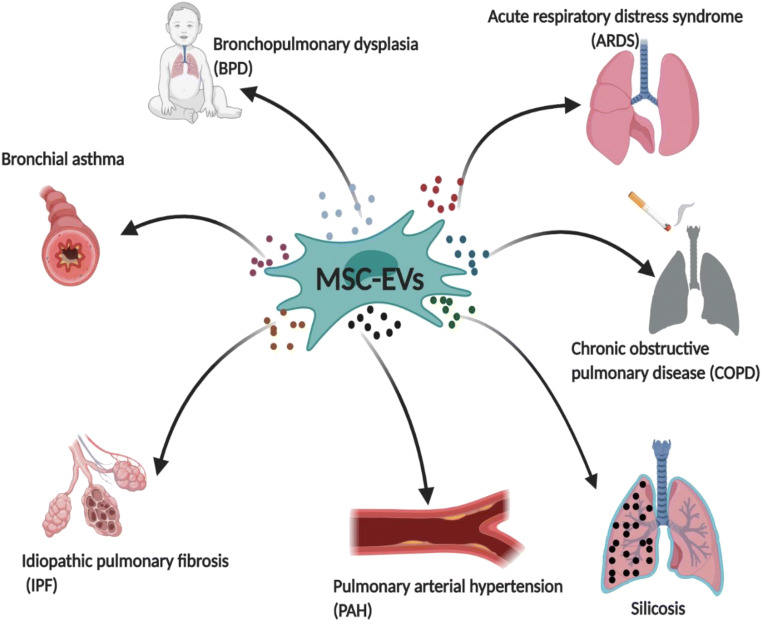

## Introduction

Respiratory diseases are leading causes of morbidity and mortality worldwide as the lung is a vital and vulnerable organ that is exposed to the ubiquity of pollutional environmental, occupational, and behavioural inhalational exposures [1, 2]. According to an analysis for the global burden of disease study (GBD) 2017, more than 500 million people in the world had a chronic respiratory disease and these diseases accounted for approximately 4 million deaths in 2017 [3]. Regarding acute lung injury (ALI), patients with ARDS occupy 10% of all beds in intensive care units (ICU) and the mortality rate for ARDS remains between 30% to 40% in most clinical research [4]. Whatever the pathophysiology of acute lung injury or chronic respiratory diseases, the overwhelming immune responses, and inappropriate reparative processes usually result in an imbalance of pro-inflammatory and anti-inflammatory cytokines, and profibrotic and anti-fibrotic factors, which give rise to irreparable damage and exert a negative impact on the quality of life [5]. The current therapies for acute lung injury and most chronic lung diseases remain in the areas of anti-inflammation and corticosteroid treatment, which have potential side effects and uncertain outcomes [6] such as increased risk of pneumonia, oral candidiasis, tuberculosis, etc. Over the past decades it has become clear that MSCs, regarded as “the Next Pillar of Medicine”, are able to restore the balance of the immune response in the process of pulmonary inflammation by modulating the cytokine network and other humoral and cellular effectors. They have been identified as having not only profound immunosuppressive effects but have also demonstrated an ability to facilitate wound healing in acute or chronic lung injury. However, mounting evidence demonstrates that only a small number of MSCs are capable of preferentially homing to damaged places and surviving for over 24 h through systematic administration [7]. Moreover, the ambiguous impacts of MSC administration, such as emboli formation and tumorigenic transformation, genetic instability, and the lack of standardized and optimized criteria contribute to the investigation of MSC-EVs as an alternative agent. Most importantly, in some experimental lung disease models, MSC-EVs obtained more effective outcomes in terms of lung vascularization and alveolarization compared to MSCs [8–10].

In this review, we pay attention to recent insights from preclinical experiments and clinical trials which have contributed to dissect the molecular mechanisms of MSC-EV effects, and highlight the existing barrier of MSC-EV application as an off-shelf agent from bench to bedside for lung diseases **(**Table 1**)**.

**Table 1:** Therapeutic effects of MSC-EVs in experimental models of lung diseases

Disease	Study	Model	MSC sourses	Route	Dose/Volume/Frequency	mechanisms
BPD	Willis et al. [40]	hypoxia-exposed mice	WJ-MSC	i.v	8.5 × 108 particles/50ul/Once	↓ alveolar simplification, fibrosis, inflammation and pulmonary vascular remodeling; ↑ total lung capcity, M2-polarized macrophges, mRNA Arg-1
BPD	Porzionato et al [8]	hypoxia-exposed mice	UC-MSC	i.t	0.64 × 1010 Evs/50ul/Once	↓thickness index for the smaller vessels,macrophage density; ↑ total number of alveoli,mean alveolar volume
BPD	Braun et al. [41]	hypoxia-exposed mice	MSC	i.p.	3.4 × 109 exosomes/50ul/Once	↓alveolarization, airspace subdivision, thickened alveolar walls, cellular infiltrates, RV hypertrophy; ↑ alveolar growth,lung blood vessel density, VEGF secretion
BPD	Chaubey et al. [42]	hypoxia-exposed mice	UC-MSC	i.p.	4.5 × 108/2.88 × 107 particles/100ul/twice	↓ alveolar injury, total cell count; inflammation, neutrophil infiltration, protein leak, septal thickness, alveolar size,PH-induced RVH, brain cell death; ↑ Myelin binding protein, TSG-6 production
ARDS	Khatri et al. [55]	influenza-induced pig	BM-MSCs	i.t	79 ± 1 μg protein per Kg/100 μl/Once	↓ virus replication, inflammation, TNF-α, CXCL10; ↑ IL-10
Severe Pneumonia	Monsel et al. [56]	*Escherichia coli*-induced mice	BM-MSCs	i.v	97 ± 90 ng protein/90ul/Once	↓ bacterial load, inflammation, lung protein permeability, monocyte phagocytosis, TNF-α; ↑ ATP levels, COX2 and IL-10 mRNA, IL-10
Acute lung injury	Hao et al. [60]	Escherichia coli-induced mice	BM-MSCs	i.v	10 × 10^9^ particles/90ul/Once	↓ MRP1 protein of monocytes, monocyte phagocytosis, PGE2/LTB4 ratio; ↑ LTB4 level; miR145 packaged in MSC-Evs
Ischemia/reperfusion injury	Li et al. [57]	hilar ligation of the left lung-induced mice	BM-MSCs	i.t	isolate from 2 × 10^6^ MSCs/30ul/Once	↓ iNOS mRNA, Caspase-3/8/9 activation,pulmonary endothelial cell apoptosis, expression of PTEN and PDCD4; ↑ Arginase-1; miR21-5p packaged in MSC-Evs
Acute lung injury	Wang et al. [62]	LPS-induced mice	AD-MSC	i.t	50 ng protein/50ul/Once	**↓** mRNA expression of iNOS, TNF-α and IL-1β, NFKB1 protein production; ↑ M2-polarization, mRNA expression of YM-1 and CD206, macrophage phagtosis;MSC-EVs transfer miR27a-3p
ARDS	Morrison et al. [67]	LPS-injured mice	BM-MSCs	i.t	MSC-EV treated macrophage 2.5× 10^5^AMs	↓ TNF-α and IL-8 secretion; macrophage phagtosis, ↑ macrophage oxidative phosphorylation; MSC-EVs transfer mitochondria to macrophages
Acute lung injury	Zhu et al. [63]	*E. coli* endotoxin-induced mice	MSC	i.t	30.9 ± 17.0 μg protein/30ul/Once	↓ Inflammatory cell influx, proetin permeability, alveolar MIP-2, extravasular lung water; ↑ KGF, IL-10
Acute lung injury	Tang et al. [64]	LPS-injured mice	BM-MSCs	i.t	isolate from 3 × 10^6^ MSCs/30ul/Once	↓ WBC, TNF-α, MIP-2 production, pulmonary capillary permeability; ↑ Ang-1 mRNA, IL-10
Lung Injury	Gennai et al. [65]	Ex vivo perfused human lung	BM-MSCs	i.v	165.6μg protein / 200ul/Once	↓ Lung weight, pulmonary artery pressure and resisitance, PH of perfusate, lactate elevation; ↑ AFC rate, lung compliance, NO in perfusate,
Severe Pneumonia	Park et al. [66]	E.coli-induced Ex vivo perfused human lung pneumonia	BM-MSCs	i.v	9.4 ± 0.2 × 10^7^particles/200ul/ Once	↓ Lung protein permeability, bacterial CFU; ↑ AFC rate and antimicrobial effect
Acute lung injury	Yi et al. [58]	LPS-injured mice	BM-MSCs	i.v	100μg protein/300ul/Once	↓ SAA3 expression; ↑ LPS-induced AEC apoptosis; miR30b-3p packaged in MSC-EVs
Sepsis	Song et al. [61]	Caecal ligation and puncture-induced sepsis mice	UC-MSCs	i.v	30μg protein/150ul/Once	↑ Survival rate, M2-polarization; MSC-EV packaged miR146a
Asthma	Castro et al. [82]	OVA-induced C57BL/6 mice	AD-MSCs	i.v	37 μg protein/50ul/ Once	↓ collagen fiber deposition, il-4/5, TGF-β, leukocyte and eosinophil counts of BALF
Asthma	Du et al. [83]	PBMCs from asthmatic patient	BM-MSCs	co-incubation	exosomes from 1 × 10^5^MSC/N/A/Once	↑ anti-inflammatory cytokine release (IL-10, TGF-β), CD4 + CD25 + Foxp3+ Tregs differenation
Asthma	Fang et al. [84]	ILC2-dominant eosinophilic mice	iPSC-MSCs	i.v	100 μg protein/20ul/Once	↓ IL-9/13 prodiction, ILC2s activation; inflammatory infiltration, eosinophils and neutrophils in BALF; miR146a-5p mediated the observed effects in allergic airway inflammation.
Asthma	Ahmad et al. [85]	Rotenone induced allergic airway in mice	BM-MSCs	i.t	1 × 10^6^MSC/N/A/Once	↓ epithelial cell stress, caspase 3/9, bronchial epithelial apoptosis; ↑ ATP level, mitochondrial complex I and IV activity; Miro1 mediated the promising effects
COPD	Harrell [94]	CS-induced mice	placental-MSCs	i.p.	N/A/0.1 ml/5 days per week (3 weeks)	↑ PaO_2_, O_2_ saturation, IL-10 secretion; ↓ PaCO_2_, pro-inflammatory cytokine production (TNFα, IL-1β, IL-12, and IFN-γ), influx of macrophages, neutrophils, NK and NKT cells
COPD	Kim [95]	porcine pancreatic elastase-induced mice	ASCs	i.t	3× 10^7^/N/A/Once	↑ ATII cell proliferation capacity, FGF2 expression; ↓ mean linear intercept
COPD	Li et al. [96]	CS-exposed rat	ips-MSCs	i.v	N/A	↓ mean linear intercept, airspace enlargement; ↑ intracellular ATP levels
COPD	Maremanda et al. [10]	CS-induced mice	MSC	i.p.	15μg protein/N/A/daily (10 days)	↓ Total cell counts, macrophage counts, neutrophil counts, CD4+ counts, KC, S100A4, PGC1α(mitochondria biogenesis), MMP9 and HMGB1
IPF	Mansouri et al. [106]	bleomycin-induced C57BL/6 mice	BM-MSCs	i.v	(8.6 ± 1 × 10^8^ particles /200ul/Once	↓collagen content, apoptotic cells, Ashcroft score, CCL2, Arg1, BAL total protein content; ↑ alveolar macrophage, nonclassical monocytes; shifting the macrophage and monocyte profiles toward that of their untreated counterparts.
IPF	Wan et al. [107]	bleomycin-induced C57BL/6 male mice	BM-MSCs	i.v	100μg MSC-EVs/N/A/Once	↓ fibroblast activation, hydroxyproline, α-SMA, collagen I, FZD6
PAH	Chen et al. [124]	MCT-induced SD rats	BM-MSCs	i.v	30μg /100ul/Once	↓ mPAP, mRVP, RV hypertrophy, pulmonary arteriole thickness index and area index
PAH	Klinger et al. [125]	Sugen5416-induced SD rats	N/A	i.v	100μg/Kg MSC-EVs/500ul/Three times	↓ right ventricular hypertrophy, muscularization of peripheral pulmonary vessels, lung macrophages; ↑ M2/M1 ratio, increased numbers of peripheral blood vessels
PAH	Zhang et al. [126]	MCT-induced rats	UB-MSC	i.v	25μg/Kg MSC-EVs/100ul/Once	↓vessel wall thickness, right ventricular hypertrophy, pulmonary vascular remodelling, PAEC apoptosis, PASMC proliferation, EndMT; ↑ Wnt5a
PAH	Lee et al. [127]	hypoxia-exposed mice	BM-MSC	i.v	10μg MSC-exosomes/100ul/Once	↓pulmonary influx of macrophages, proinflammatory and proproliferative mediators (MCP-1, HIMF), STAT3 activation; miRNA-17 superfamily, miR-204
PAH	Zhang et al. [128]	MCT-induced SD rats	ASCs	i.p	N/A	↓ RVSP, RV/(LV + S), MT + IT, CSA; ↑ proliferation of HPAECs; miR191 packaged in ASC-EVs accelerated HPAEC proliferation through BMPR2
PAH	Liu et al. [129]	MCT-induced SD rats	BM-MSC	i.v	30μg MSC-microvesicles/500ul/Once	↓ PAP, RVSP, pulmonary vessel wall thickness index, pulmonary vessel lumen area index, inflammation score, collagen fiber volume fraction; ↑ ACE2 mRNA in lung, plasma levels of Ang-(1–7)
PAH	Hogan et al. [130]	hypoxia-exposed mice	BM-MSC	i.v	2*10^7^ particles /200ul/Once	↓ lactate, mitochondrial damage; ↑ amino acid metabolism, glucose oxidation, OCR, mitochondrial metabolism, PDH, GLUD1
Silicosis	Bandira et al. [138]	MCT-induced SD rats	BM-MSCs	i.v	100μg /100ul/Once	↓ mPAP, mRVP, RV hypertrophy, pulmonary arteriole thickness index and area index
Silicosis	Pinney et al. [139]	silica-induced mice	BM-MSCs	i.v	40μg MSC-EVs/500ul/Once	↓ Ly6Chi monocyte infiltration, inflammatory mediators (TNF-, IL-6), silicotic nodules, hydroxyproline accumulation
Silicosis	Chio et al. [140]	silica-induced BL/6 J mice	BM-MSCs	i.v	10μg /100ul/Once	↓ wet/dry ratio, total BAL cells, foamy macrophages/total macrophages, inflammation response, collagen

## MSC-EVs: Definition, Characteristics and Potential Effects in Lung Diseases

MSC-EVs are round signal molecules delimited by a lipid bilayer membrane, which play a prominent role in extracellular communications through delivering parental cell-derived active cargos such as bioactive proteins, mRNAs, noncoding RNAs and organelles to recipient cells [11]. Based on the Minimal information for studies of Extracellular Vesicles 2018 (MISEV2018), the International Society for Extracellular Vesicles (ISEV) suggests a new nomenclature of MSC-EVs subtypes, which is based on: 1) physical characteristics of EVs; 2) biochemical composition; or 3) descriptions of conditions or cell of origin [12]. However, most studies currently still use the general classification for MSC-EV subtypes: exosomes (30-120 nm), microvesicles (100-1000 nm), and apoptotic bodies (800-5000 nm) [13]. Recently, research on EV biogenesis and shedding has indicated that exosomes and microvesicles have two different secretory mechanisms, of which exosomes are derived and generated through endocytic pathway, and subsequently fuse either with lysosomes or with plasma membrane [14, 15]. By contrast, microvesicles are originated by plasma membrane budding and release directly from the cell surface [16]. Furthermore, the contents of the EV cargo are dependent on the type of their parental cells and the microenvironment of the releasing cells [17, 18].

The lungs are the primary organ of the respiratory system that are in contact with the external environment containing pathogens and microbes. Pulmonary homeostasis is maintained by the communication between local stromal cells and resident immune cells that sense the dynamic microenvironment. Upon the disruption of homeostasis by risk pathogens, resident macrophages as the first line of defense against various pathogenic microorganisms and the primary source for the release of proinflammatory cytokines and chemokines, such as TNF-α, IL-1β, and macrophage inflammatory protein-2(MIP-2), recruit neutrophils and monocytes respectively to propagate the immune responses [19]. In an extremely uncontrolled microenvironment, excessive inflammation, aberrated immunomodulation or unknown etiologies call for clinical intervention. To date, antibiotics, corticosteroid, and invasive ventilation are primary choice for treating respiratory disease, but multi-drug resistance, opportunistic infection, and unrecoverable injury are main side effects to patients. In recent years, compared to the pharmacological treatments, MSC-EVs have exhibited immunosuppressive and reparative properties in the way of low immunogenicity, long half-life, in vivo stability, and high delivery efficiency, which also contribute to attenuate lung injury and facilitate wound closure. Accordingly, employing MSC-EVs is likely to be a promising approach due to their ability to reduce lymphocyte infiltration and pro-inflammatory cytokine secretion, inhibit bacteria or virus replication, regulate endothelial and epithelial permeability, and promote tissue repair [20–22]. Moreover, accumulating data have shown MSC-EVs are capable of modulating proliferation, maturation, polarization, and migration of different immune effector cells depending on the context of delivering various cytokines, transcription mediators, and organelles, which contribute to the preferential characteristics of MSC-EVs in their immunomodulatory effects [23, 24].

## Molecular Mechanisms of MSC-EVs in Lung Diseases

### Bronchopulmonary Dysplasia

Bronchopulmonary dysplasia (BPD) is a chronic respiratory disease most commonly seen in preterm infants and neonates who require mechanical ventilation and oxygen therapy for acute respiratory distress [25], characterized by a dysregulated immune response, decreased numbers of alveoli and blood vessels, and dysfunction of the aveolar-capillary membrane [26]. In preclinical studies, newborn mice or rats exposed to a hyperoxia (75%) microenvironment are widely used to mimic the pathogensis of human BPD [27]. Over the years, systematic administration, or local injection (intranasal [28] or intratracheal [29]) of MSCs have defined the beneficial impact on attenuating experimental BPD through inhibition of *N*-methyl-D-aspartic acid (NMDA) receptors [30], renin-angiotensin system (RAS) [31], TLR4 expression [32], decorin [29] and CTGF secretion [33], accompanied by upregulating the production of aminoacyl-peptide hydrolase [34], PTX3 [35], VEGF [33], stromal cell-derived factor 1 [36], macrophage stimulating factor 1 [37], and osteopontin [37], leading to increased survival rate, downregulated inflammation- and hyperoxia-induced defective alveolarization, and reduced lung fibrosis in experimental BPD mice. Moreover, MSC stably transfected with a truncated version of CC chemokine ligand 2 (CCL2) promotes macrophage activation, and is seen to be more effective than MSCs alone [38]. These promising preclinical data have contributed to the application of MSCs in clinical trials **(**Table 2**)**. For example, Chang et al launched a phase I dose-escalation trial (NCT01297205) of hUCB-derived MSC transplantation in BPD, recruiting 9 preterm infants of which three were given a low dose (1*10^7^ cells/Kg) and the other six were administrated a high dose (2*10^7^ cells/Kg). Both groups showed that MSC administration in treating BPD in preterm infants is safe and feasible [39].

**Table 2:** Characteristics of MSC treatments in lung diseases (completed clinical trials)

Clinical Trial ID	Lung Diseases	Name of Clinical Trial	Phase	MSC type	Dose	Frequency	Route	Patients enrolled	Follow up
NCT03601416	BPD	Human mesenchymal stem cells for moderate and severe bronchopulmonary dysplasia	Phase 2	UC-MSC	2.5/5 million cells/kg	once	i.v.	48(24/24)	24 months
NCT01297205	BPD	Safety and efficacy evaluation of PNEUMOSTRM treatment in premature infants with bronchopulmonary dysplasia	Phase 1	UC-MSC	10/20 million cells/kg	once	i.t	9(3/6)	12 weeks
NCT01632475	BPD	Follow-up study of safety and efficacy of PNEUMOSTRM in premature infants with bronchopulmonary dysplasia	Phase 1	UC-MSC	10/20 million cells/kg	once	i.t	9(3/6)	24 months
NCT01902082	ARDS	Adipose-derived mesenchymal stromal cells in acute respiratory distress syndrome	Phase 1	AD-MSCs	10 million/kg	once	i.v.	20(10/10)	28 days
NCT01775774	ARDS	Human mesenchymal stromal cells for acute respiratory distress syndrome (START)	Phase 1	BM-MSC	1,5,10 × 106/kg	once	i.v.	9(3/3/3)	12 months
NCT02097641	ARDS	Human mesenchymal stromal cells for acute respiratory distress syndrome (START)	Phase2a	BM-MSC	1 × 107/kg	once	i.v.	60(40/20)	12 months
NCT02611609	ARDS	A Phase 1/2 Study to Assess MultiStem® Therapy in Acute Respiratory Distress Syndrome(MUST-ARDS)	Phase 1/2	MultiStem®	300/900million	once	i.v.	30(10/10/10)	12 months
NCT02095444	H7N9-ARDS	Using human menstrual blood cells to treat acute lung injury caused by H7N9 bird flu virus infection	Phase1/2	MB-MSCs	1 × 106/kg	4times; twice/week	i.v.	61(17/44)	60 months
ChiCTR2000029990	COVID-19	Clinical trials of mesenchymal stem cells for the treatment of pneumonitis caused by novel coronavirus pneumonia (COVID-19)	Phase1/2	ACE2- MSCs	1 × 106/kg	once	i.v.	10(7/3)	N/A
NCT00683722	COPD	Prochymal (human adult stem cells) for the treatment of moderate to severe chronic obstructive pulmonary disease (COPD)	Phase 2	MSC	100 × 10^6^ cells	once	i.v.	62(30/32)	24 months
NCT01872624	COPD	Safety study of bone-marrow derived mesenchymal stromal cells associated with endobronchial valves in emphysema	Phase 1	BM-MSC	100 × 10^6^ cells	once	i.v.	10	4 months
NCT01306513	COPD	Safety and feasibility study of administration of mesenchymal stem cells for treatment of emphysema	Phase 1	BM-MSC	each patient varied	twice	i.v.	10	17 weeks
12,614,000,731,695	COPD	Mesenchymal stromal cell infusion modulates systemic immunological responses in stable COPD patients	Phase 1	BM-MSC	2.0 × 106/kg	twice	i.v.	9	12 months
EHD33/1SC/16-02-2010	PAH	Stem cell therapy for idiopathic pulmonary fibrosis: a protocol proposal	Phase 1b	AD-MSCs	0.5/1.0 × 106/kg	three times	i.v.	14	12 months
NCT01385644	PAH	A study to evaluate the potential role of mesenchymal stem cells in the treatment of idiopathic pulmonary fibrosis (MSC in IPF)	Phase 1b	Placental MSC	10/20 million cells/kg	once	i.v.	8(4/4)	6 months
NCT02013700	PAH	Allogenic human cells (hMSC) in patients with idiopathic pulmonary fibrosis via intravenous delivery (AETHER)	Phase 1	BM-MSC	20 million	once	i.v.	9	60 weeks

**Fig. 1:**
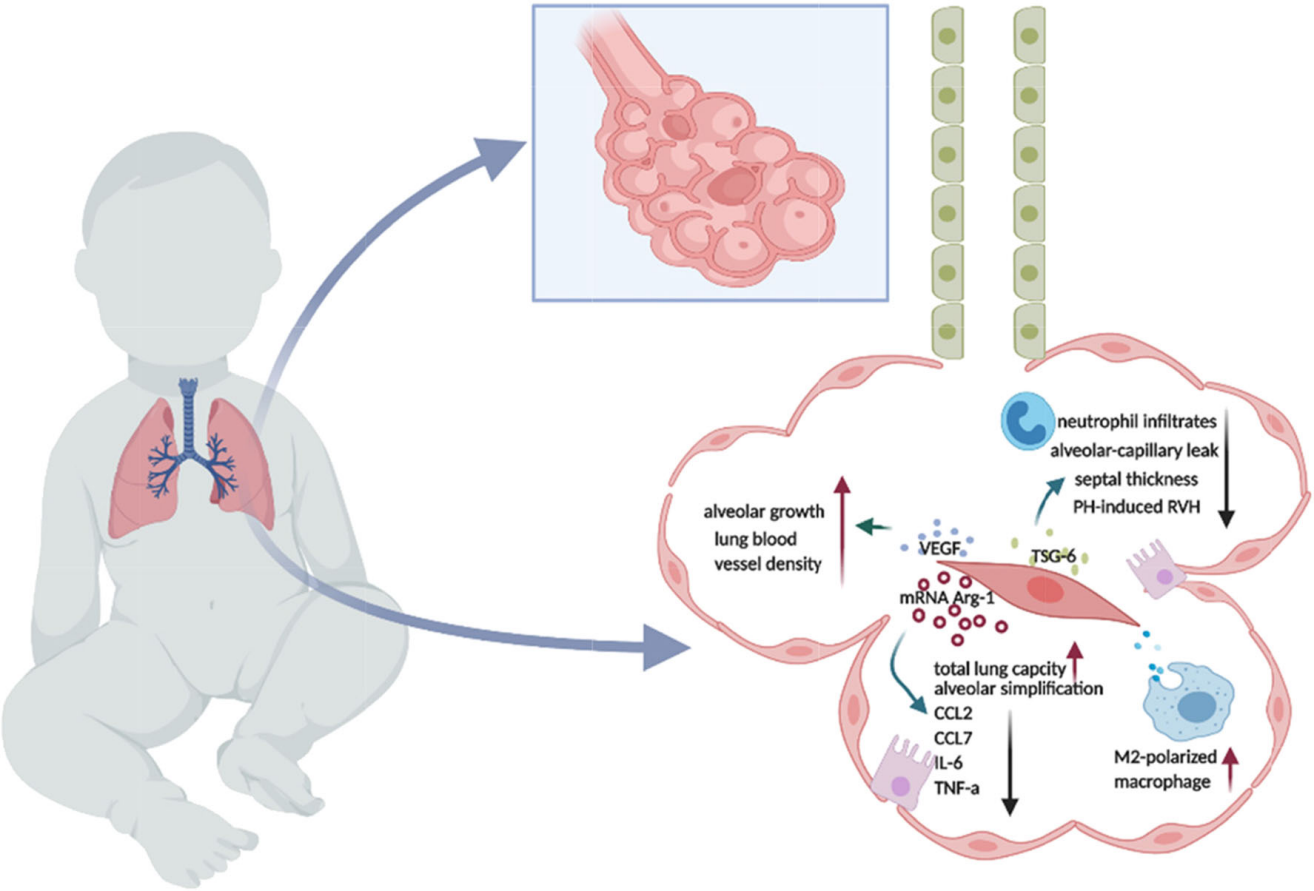
Therapeutic effects of MSC-EVs in BPD. MSC-exosomes deliver biological molecules to modulate macrophage polarization, shifting into an anti-inflammatory (M2) phenotype. TSG-6 packaged in MSC-EVs downregulates neutrophil infiltrates, alveolar-capillary leak, septal thickness, and PH-induced RVH. Arg-1 mRNAs wrapped in MSC-EVs promote total lung capacity and alveolar simplification and decrease the production of pro-inflammation cytokines (CCL2, CCL7, IL-6, TNF-α). Meanwhile, MSC-EVs deliver VEGF to damaged tissue to regulate alveolar growth and lung blood vessel density.

Nevertheless, safety concerns regarding the transplantation of MSC in newborns have facilitated the investigation of MSC-EV effects in BPD. Willis et al conducted pioneering research to assess the efficacy of MSC-exo treatment in an experimental hyperoxia-induced BPD model and to investigate mechanisms underlying the therapeutic effect [40]. They have demonstrated that MSC-exos administrated intravenously at the concerntration of 8.5*10^8^ particles/50ul improve pulmonary development, ameliorate septal fibrosis, restore lung architecture, and enhance peripheral pulmonary arterial remodeling through macrophage phenotype modulation [40]. Noteworthily, the other studies done by Braun and Porzionato et al have also shown that MSC-EV injection intraperitoneally(3.4*10^9^/50ul) or intratracheally(6.4*10^9^ EVs/50ul) increases blood vessel number and lung size, prevents right heart hypertrophy, and inhibits alveolar growth disruption via anti-inflammatory and pro-angiogenic mechanisms [8, 41]. Moreover, MSCs transferred exosomal factor-TSG-6 partially restores the alveolar-capillary leakage, increasing chord length and alveolar simplification in hypoxia-induced neonatal BPD mouse models [42].There is an ongoing clinical trial (NCT03857841) aiming to investigate the intravenous infusion of BM-MSC-drived EV (UNEX-42) on preterm neonates at high risk for BPD. This interventional, randomized, and placebo-controlled phase I clinical trial will recruit 18 infants and has three dose arms: 20/40/60 pmol phospholid/Kg body weight. Collectively, MSC-EV administration holds great therapeutic potential for BPD by facilitating macrophage polarization, improving alveolarization and angiogenesis, and reducing collagen density in the experimental studies of BPD. **(**Fig. 1**).**

### Acute Respiratory Distress Syndrome and Severe Pneumonia

Acute Respiratory Distress Syndrome (ARDS) is a form of severe hypoxemic respiratory failure caused by several risk factors, such as pneumonia, sepsis, and trauma, which is characterized by diffuse alveolar damage (DAD) with apoptosis of alveolar type I and II cells, accumulation of proteinaceous oedema, and hyaline membrane formation in the alveolar space [43]. Since the definition of ARDS was established 50 years ago, there has been remains no specific pharmacological treatment for ARDS. Data from preclinical experiments have shown that MSCs prevent the development of ARDS in vitro and in vivo in the experimental acute lung injury (ALI)/ARDS mice models which are instilled with lipopolysaccharide (LPS) or bacteria. MSCs have been reported to secrete various kinds of paracrine factors to restore epithelial and endothelial cell permeability (Ang1, IL-1ra, PGE2, HGF) [44–46], facilitating macrophage phagocytosis (IL-6, PGE2) [47], downregulating acute inflammation (IL-1ra, TSG-6, IGF-1, Lipoxin A4) [48], and improving alveolar fluid clearance (KGF7) [49, 50]. Importantly, early clinical trials (phase I and phase II a/b) suggest that it is safe to give MSCs to patients with ARDS [51], and the MUST-ARDS study conducted by Athersys Inc. with a patented bone marrow-derived adult multipotent progenitor cell product (MultiStem) reported a significant reduction in 28-day mortality accompanied by an increase in both ventilator and ICU free days in patients who had received cell therapy [52]. More recently, systematic MSC administration has shown its outstanding properties in improving clinical symptoms and modulating immune responses in critically ill COVID19-ARDS patients [53, 54].

Similarly, MSC-EVs have been shown to be beneficial to experimental ARDS in viral- (H5N1, H1N1), bacterial-(*Escherichia coli*.), and LPS-induced acute lung injury (ALI). In an influenza A (H5N1)–induced ALI, umbilical cord derived MSC-exosomes at the concentration of 1*10^10^ particles/90ul have been shown to be more effective in improving alveolar fluid clearance and attenuating protein permeability of alveolar epithelial cells than UC-MSCs due to their greater production of Ang1 and HGF. Moreover, PKH-26-labeled MSC-EVs are able to merge with epithelial cells to suppress virus replication, virus shedding, virus-induced apoptosis, and hemagglutination activity in the other influenza-induced ALI porcine model [55]. Additionally, in an *E.coli* pneumonia-mediated ALI murine model, Monsel and colleagues have elucidated that human MSC-derived microvesicles (MVs) administration (97 ± 90 ng protein/90ul) decreases the influx of inflammatory cells, and the level of cytokines, protein, and bacteria, coupled with the increased intracellular ATP production in damaged alveolar epithelial type 2 cells, partially through KGF secretion [56]. Most of the studies used an endotoxin (LPS)-induced animal model to mimic the human ARDS/pneumonia microenvironment and to evaluate the MSC-EV effects. Noteworthily, miRNAs packaged in MSC-EVs have been found to play a key role in attenuating ARDS lung injury. It has been reported that MSC-EVs delivered miR21-5p [57]/miR30b-3p [58]/ miR100 [59]/ miR145a [60] /miR146a [61] to attenuate the inflammatory responses. Moreover, MSC-EVs also transferred miR27a-3p / miR146a to modulate macrophage polarization [59, 62]. Besides miRNAs, Zhu et al and Tang et al have demonstrated that MSC-EVs (30.9 ± 17.0μg protein/30ul or isolated from 3*10^6^ MSCs/30ul) are capable of reducing inflammatory cell influx, protein permeability, MIP2 production, extravascular lung water, and increasing IL-10 secretion in the LPS-induced mouse models [63, 64]. More importantly, in the ex vivo perfused human lung models, Gennai et al and Park et al have shown that MSC-microvesicles (16.5μg/200ul or 9.4 ± 0.2*10^7^ particles/200ul) are able to decrease pulmonary artery pressure and resistance, lactate elevation, bacterial CFU, lung protein permeability and lung weight, and increase alveolar fluid clearance (AFC) rate, lung compliance and antimicrobial effect [65, 66]. MSCs also donate functional mitochondria to macrophages to modulate macrophage polarization through enhancement in oxidative phosphorylation and to improve mitochondria function of epithelial cells, resulting in wound closure in a clinically relevant model of ARDS [67, 68].

A pilot clinical trial (NCT04276987) regarding aerosol inhalation of allogenic adipose MSC-exosomes for treating COVID in critically ill patients has been completed by Rujin Hospital, Shanghai. This forerunning phase I trial recruited 24 participants who received conventional treatment and five times aerosol inhalation of MSC-exosomes (2*10^8^ nano vesicles at day1,2,3,4,5), but no results have been posted to date.

### Bronchial Asthma

Bronchial asthma is one of the most common and chronic lung diseases in children and adults, whose pathophysiology is underpinned by a chronic inflammation of the airway walls accompanied by mucus hypersecretion, epithelial shedding, metaplasia and hyperplasia of goblet cells, increased collagen deposition, and hypertorophy and hyperplasia of airway smooth-muscle [69, 70]. The current treatments for asthmatic patients are largely symptomatic and ineffective, and diverse side effects of these therapies has led researchers and clinicians to seek safe and effective candidates for this chronic disease. Studies into the effect of MSC on bronchial asthma have shown great potential in attenuating the major pathologic characteristics of asthma including airway immune responses, hyperresponisveness, and remodelling.

Boldrini-Leite et al have found that in the ovalbumin (OVA)-induced asthma BALB/c mice model, the MSC-treated group is able to reduce the amount of eosinophil, lymphocyte, total protein, H_2_O_2_, IL-5, IL-13 and IL-17a in the BALF [71]. Similarly, Abreu et al and Song et al have demonstrated that MSCs are capable of reducing lung inflammation and tissue remodeling through promoting the production of anti-inflammatory cytokines and angiogenic factors (IL-4, IL-13, TGF-β, VEGF, VCAM-1, ICAM-1) [72, 73], activating TGF-β signaling to induce M2-like macrophage polarization [74], decreasing oxidative stress, the thickness of basement membrane, epithelium, subepithelial and smooth muscle layer as well as the number of mast cells and goblet cells [75–77]. Moreover, in house dust mite-induced allergic asthma, MSCs attenuate the secretion of epithelial cell-derived alarmins IL-ra, pro-Th2 cytokine IL-25, and the number of activated and antigen-acquiring CD11c + CD11b + dendritic cells [78, 79]. Additionally, MSC-conditional medium has also shown beneficial effects in decreasing pathologic scores of the OVA-injuried lung by elevating the mRNA expression of T-bet and IFN-γ, while decreasing the GTAT3 mRNA expression [80]. Unfortunately, although the preclinical evidence has shown the beneficial effects in asthema and several clinical trials in asthmatic patients are ongoing, there remains no data of clinical trials published to date [81].

Compared to MSCs, MSC-EVs (50ul) isolated from 1*10^5^ AD-MSCs show a similar capacity to reduce lung inflammation and a reversal of injured tissue remodelling in the OVA-induced (20μg) asthmatic model by reducing the amounts of eosinophils, collagen fiber in airways, TGF-β production in lung tissue, and CD3 + CD4+ T cells counts in the thymus [82], and also promote the proliferation and immunosuppressive ability of Treg cells [83]. Fang and colleagues have demonstrated that MSC-sEVs (2 × 10^10^ sEVs/100μg protein) exhibit a significant impact in inhibiting inflammatory cell infiltration, airway hyper-responsiveness, mucus secretion, and downregulating T help 2 cytokines and the function of group 2 innate lymphoid cells (ILC2s), More specifically, miR146a-5p packaged in the sEVs has been revealed to mediate the above effects to rejuvenate ILC2s-dominant allergic airway injury [84]. In the rotenone (Rot)-mediated (0.3 mg/kg) airway injury and allergic airway murine model, Miro1, a mitochondria Rho GTPase 1, is capable of facilitating mitochondria transfer from MSCs to damaged epithelial cells to reverse mitochondria dysfunction [85]. Altogether, MSC-EVs serve as important mediators which target on the immunomodulation and airway reconstruction, but their mechanisms of action are still under investigation.

### Chronic Obstructive Pulmonary Disease (COPD)

COPD is a common, preventable, and treatable lung disease that is characterized by mucous hypersecretion and ciliary abnormalities, airflow obstruction and hyperinflation, gas change dysfunction and pulmonary hypertension, which damages airways (bronchitis-bronchiolitis) and alveoli (emphysema), leading to chronic inflammatory responses, persistent respiratory symptoms and airflow limitations [86, 87].

Cigarette smoke (CS) has been recognized as the foremost risk factor contributing to COPD development. The global strategy for the diagnosis, management, and prevention of COPD 2017 (GOLD2017) elucidated that COPD is projected to be the third leading cause of death worldwide by 2020 [88]. Current pharmacological agents are are poorly responsive to the disease’s progress and mortality [88]. Based on a growing body of evidence, it has been clear that MSCs have brought hopeful and promising effects in COPD. Shigemura et al were the first to establish that adipose tissue-derived stromal cells (ASCs) secrete HGF to repair pulmonary emphysema, and improve gas exchange and exercise tolerance [89]. Simultaneously, MSCs also modulate gene expression profiles of adjacent cells to reduce airway inflammation and restore alveolar architecture. In addition, Kim and colleagues have shown that compared to a control group, 834 genes were differentially expressed after human cord blood-derived-MSC administration in a smoke-induced COPD mouse lung model, more specifically, the genes (Hbb and Hba) with oxygen transport and antioxidant functions are significantly increased on day 1 and day 14 [90]. In an elastase-induced emphysema model, MSC administration facilitates the protease/anti-protease balance and decreases the activity of matrix metalloproteinase 9 [91]. Additionally, MSCs overexpressed with the all-trans retinoic acid (ATRA) combined with p-70S6 kinase-1 (*p70S6k1*) enhances the therapeutic effects in elevating static lung compliance, alveolar surface area, and decreases mean line intercepts [92]. A large amount of promising results contribute to MSC application in COPD patients. To the best of our knowledge, the first clinical trial (NCT00683722) was initiated by Weiss and colleagues, which is a Phase II, multicentre, randomized, double-blind placebo-controlled study, study involving sixty-two patients with GOLD stage II and III COPD patients. After a 2-year follow up, no infusional toxicities or serious adverse events related to MSC administration were deemed to have happened, and a significant decrease in C-reactive protein (CRP) level was observed in MSC-injected patients [93].

**Fig. 2:**
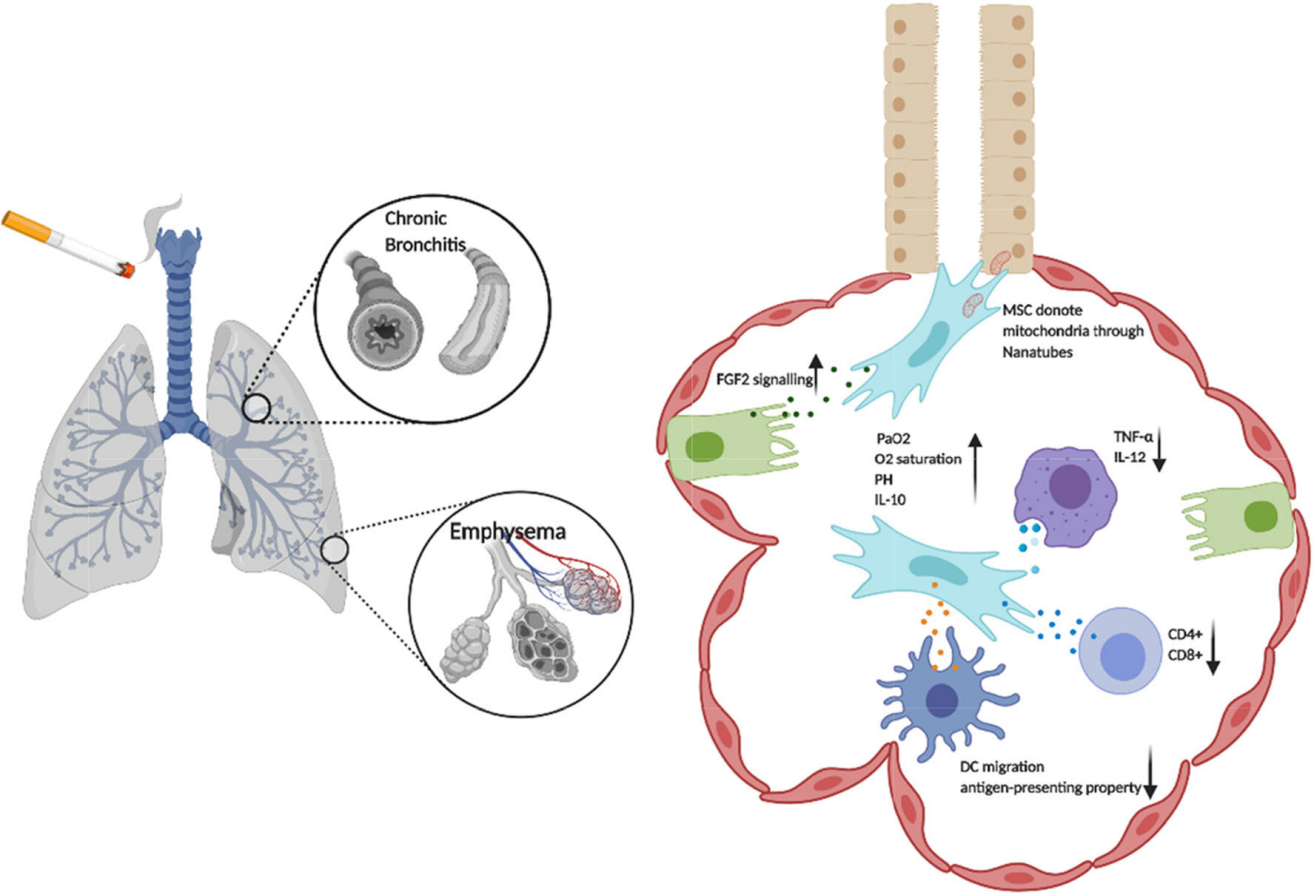
Role of MSC-EVs in COPD. MSC-EVs elevate O2 saturation, pH, PaO2, IL-10 secretion, and decrease pro-inflammatory cytokine production (TNF-α, IL-12). Functional mitochondria are donated by MSCs to bronchial epithelial cells through nanotubes, contributing to enhance oxidative phosphorylation of the targeted cells. MSC-EVs uptake by Alveolar type II cells stimulate FGF2 intracellular signalling activation. MSC-EVs also downregulate the pro-inflammatory cytokine secretion (TNF-α, IL-12), deactivate CD4+/CD8+ T cells, and suppress the migration and antigen presenting property of dendritic cells.

MSC-EVs have also exhibited protective effects in experimental COPD. Based on a chronic CS-induced COPD mice model, MSC-exosomes were shown to significantly improve lung function, including elevated O2 saturation, pH, PaO2, IL-10 secretion, and to decrease pro-inflammatory cytokine production (TNF-α, IL-12), the total number of lung-infiltrated macrophages, the capacities of antigen-presenting alveolar macrophages, IL-17A producing-NK/NKT cells, neutrophils to attenuate inflammation. Moreover, the exosomes are capable of affecting the migratory and antigen-presenting properties of DCs, which contribute to the attenuated activation of CD4+ and CD8+ T lymphocytes. Importantly, this study has also demonstrated that inhalation of MSC-exosomes indicate an improved FEV1, PEF, 6-min walking distance (6MWD) and quality of life in COPD patients, with alleviated emphysematous changes, including less hyperexpanded lung, less flattened diaphragms and reduced centrilobular and paraseptal emphysema [94]. In what could be another mechanism for MSC-EVs in COPD, Kim et al have shown that unlike MSC-derived natural exosomes, MSC-derived artificial nanovesicles (3 × 10^7^ artificial nanovesicle particles generated from 7 × 10^7^ ASCs) display a more efficient regenerative capacity to reduce the mean linear intercept (MLI) primarily through activating the FGF-2 signalling pathway [95]. In addition, functional mitochondria packaged in EVs have recapitulated MSC effects in preclinical models of COPD. iPSC-derived MSCs transfer their mitochondria to human bronchial epithelial cells through tunnelling nanotubes to alleviate CS-induced damage and to rescue the dysfunctional mitochondria of human airway smooth muscle cells in rats [9, 96]. Maremanda et al have demonstrated that MSC-exosomes also modify mitochondrial genes in bronchial epithelial cells, including enhancing fusion gene expression (mfn1, mfn2, and opa1) [10] **(**Fig. 2**)**. To date, MSC-EVs, as a new frontier, have provided convincing evidence of positive effects in COPD by modulating chronic inflmammtion, inhibiting emphysema, and restoring dysfunctional mitochondria.

### Idiopathic Pulmonary Fibrosis

Idiopathic pulmonary fibrosis (IPF) is defined as a chronic, degenerative and progressive lung disease characterized by alveolar epithelial cell dysfunction, fibroblast proliferation, extracellular matrix collagen accumulation, and interstitial inflammation, leading to exertional dyspnoea, dry cough, weight loss malaise and arthralgia [97]. The comprehensive understanding of IPF pathogensis and effective treatments remains elusive. Available data have shown that MSC administration restores a bleomycin-induced lung injury model with a reduction in inflammatory response and collagen deposition, and an improved Ashcroft score [98, 99]. Reddy and colleagues have demonstrated that MSCs are capable of ameliorating the expression of pro-inflammatory (IL-1b, TNF-β, etc.), pro-fibrotic (bFGF, CTGF, etc) transcripts in injured lungs, and maintaining MMP-TIMP balance [100], correcting the inappropriate epithelial-mesenchymal relationships through stanniocalcin-1 to ameliorate oxidative stress and endoplasmic reticulum stress [101]. Moreover, Akram et al have reported that MSC-CM promotes human small airway epithelial cell (SAEC) wound repair by secreting an array of proteins (Fibronectin, Lumican, Periostin, IGFBP) [102], increasing the amount of Tregs, decreasing cytotoxic T cells coupled with a concomitant suppression in α-smooth muscle actin (α-SMA) [103], contributing to a reduced hydroxyproline (HYP) deposition, myeloid differentiation primary response gene 88 (MyD88), and TGF-β signaling activation [104]. Recently, Gad et al. have shown that the therapeutic anti-fibrotic properties of MSCs are mediated through the inhibition of SMAD-3/TGF-β signalling [105].

Unfortunately, research into MSC-EV effects on IPF is limited. Mansouri and colleagues have presented that MSC-EVs are able to reduce the degree of whole lung apoptosis, reverting pulmonary fibrosis, improving the Ashcroft score, and increasing the number of alveolar macrophages (CD206) and nonclassical monocytes. Additionally, bioinformatics analysis has revealed that eighty-four peptides varied significantly between MSC-EV treatment and fibroblast-derived EV treatment with myeloid/monocyte cells [106]. More recently, Wan et al have conducted research on MSC-EV effects on pulmonary fibroblasts, and have demonstrated that the EVs suppress fibroblast proliferation, migration, invasion, and differentiation in IPF, confirmed by Cell Counting Kit (CCK-8), Transwell assay, and gain- and loss-of-function assays through overexpressing miR-29-3p [107].

### Pulmonary Arterial Hypertension

Pulmonary arterial hypertension (PAH) is a chronic and devastating disease in which extensive obliterative changes are associated with elevated pulmonary arteries pressure, pulmonary vascular resistance, and right ventricular (RV) dysfunction, resulting in vascular fibrosis and stiffening [108, 109]. Mounting evidence has shown that treatment with rat MSC or human MSC treatment is able to decrease pulmonary vascular resistance, improve vascular endothelial function and right ventricular function in the monocrotaline or Su5416/hypoxia-injured lung [110–113] through regulating [Ca^2+^]i signal-associated cellular behaviours [114], normalizing the expression levels of apoptosis (active-caspase-3), cellular proliferation (p-38 MAPK and ERK5), and inflammation markers (TNF-α, IL-1β, IL-6) [115], suppressing TLR-4 signalling [116], expressing Heme Oxygenase-1 (HO-1), enhancing let-7a expression [117], and dampening endothelial-mesenchymal transition (EndMT) [118, 119]. Moreover, high throughput sequencing has demonstrated that six miRNAs of MSCs (upregulated: miR573 and miR1246; downregulated: miR206, miR-133a-3p, miR-141-3p and miR-200a-3p) are differentially expressed with co-culturing with human pulmonary arterial endothelial cells (HPAECs) [120]. Simultaneously, there have been several clinical trials completed aiming at the MSCs effects on IPF patients. Tzouvelekis and colleagues conducted a prospective, non-randomized, no placebo-controlled, phase 1b clinical trial (EHD33/1SC/16-02-2010) to investigate the safety of MSCs in IPF, which showed that MSC administration is an acceptable safe treatment regimen to IPF. However, this study did not deteriorate in functional parameters and indicators of quality of life [121]. Similarly, the other dose-escalation phase 1b trial (NCT01385644) has indicated there is no significant change in forced ventilation capacity (FVC), diffusing capacity of the lungs for carbon monoxide (DLCO), 6MWD and CT fibrosis score of the IPF patients compared with baseline after 6 months [122]. Nevertheless, the AETHER trial (NCT02013700), the allogeneic human MSCs in patients with IPF via intravenous delivery, has demonstrated a 3.0% decline in % predicted FVC and 5.4% mean decline in % predicted DLCO. More recently, the first-in-human high-cumulative-dose stem cell therapy in IPF patients was conducted by Averyanov and colleagues. Twenty patients with a current FVC ≥ 40% of predicted and DLCO ≥20% with a lung function decline (FVC and DLCO) ≥10% over the last 12 months were recruited into a phase I/IIa study, and received two intravenous doses of MSCs (2*10^8^ cells) every three months (total amount: 1.6*10^9^ cells). After the study was completed, no significant adverse effects were found in the MSC-administrated group, and they were observed having a better outcome for the 6MWD, for DLCO in 26 weeks, and for FVC in 39 weeks compared with the placebo group [123].

Intravenous injection of MSC-EVs in the monocrotaline-induced PH (MCT-PH) rat model has indicated similar effects to those of MSCs in ameliorating the mean pulmonary artery pressure (mPAP), mean right ventricle pressure (mRVP), RV hypertrophy, the pulmonary arteriole area index (AI) and the thickness index (PI) [124], enhancing macrophage polarization [125], and deactivating EndMT [126]. According to the research investigating paracrine mechanisms, MSC-EVs are capable of suppressing the signal transducer and activator of transcription 3 (STAT3), and upregulating the miR17 superfamily and miR204 in the context of PAH rats [127]. In addition, MSC-exosome packaged miR191 restores monocrotaline pyrrole (MCTP)-induced lung injury by repressing bone morphogenetic protein receptor 2 (BMPR2) [128]. Moreover, microvesicles derived from MSCs promote ACE2 mRNA and plasma levels of Ang- (1–7) in the injured lung [129], and MSC-exosome administration increases the expression of pyruvate dehydrogenase (PDH) and glutamate dehydrogenase 1 (GLUD1), leading to improved mitochondrial health in the hypoxia-induced PAH mouse model [130]. However, even though many promising data support MSC therapeutic effects for PH, to date, no clinical trials in this area can be found in “*ClincalTrials.gov*”.

### Silicosis

Silicosis is a preventable but chronic, progressive, and fatal occupational respiratory disease caused by the long-term inhalation of respirable crystalline silica dust, which lacks specific pharmacological treatment and potentially increases the morbidity of pulmonary tuberculosis [131]. Mounting evidence has shown that MSC transplantation contributes to a remissive effect on silica-induced lung fibrosis [132, 133] through decreasing the level of Caspase-3 protein [134], downregulating the expressions of fibrosis marker proteins (Vimentin and α-smooth actin) [135], the mRNA levels of collagen I, collagen III, and fibronectin, and the secretion of TGF-β and hydroxyproline [136], in parallel with elevating the ratio of Bcl-2/Bax, the expression of epithelial marker protein (E-cadherin, cytokeratin19). Due to the low prevalence rate, only one clinical trial (NCT01977131) was found to assess the MSC effect in patients with pulmonary silicosis. Autologous BM-MSCs transfected with human HGF cDNA (MSCs/HGF) were administrated intravenously at a dose of 2*10^6^/kg in four patients. It has been found that MSCs/HGF were capable of ameliorating the symptoms of cough and chest distress, and improving pulmonary function. Moreover, in the labtorary tests of peripheral blood from the patients, the ratio of the peripheral CD4+/CD8+ was increased, and serum IgG levels were decreased [137].

MSC-EVs have also shown a therapeutic effect on silica-induced experimental silicosis as a cell replacement of MSCs. Bandeira et al have stated that MSC-EVs lead to a reduction in collagen fiber content, lung static elastance, size of granuloma, and the number of macrophages inside granuloma and in the alveolar sept in the silica intratracheal instillation of C57BL/6 mice [138]. Moreover, intravenous administration of human MSC-exosomes is capable of reducing the extent of Ly6C^hi^ monocyte infiltration into the injured lung, the size of silicotic nodules, the total number of white cells in BALF, and the expression of inflammatory (TNF-α, IL-6) and pro-fibrotic genes (COL1A1) in the lung tissue [139]. However, Choi et al have shown that even though MSC-microvesicles present beneficial effects to silica-mediated silicosis, their therapeutic efficiency is less than that of MSC transplantation [140]. Finally, it has become clear that MSCs or MSC-EVs have shown therapeutic potential to pulmonary silicosis, but dissecting the compensive biological mechanisms is the next milestone for the application in clinicial work.

## Challenges in MSC-EV Application in Clinics

Despite the wealth of promising preclinical results for MSC-EV application in lung diseases, unfortunately, until now there have been only two clinical trials in regard to evaluating MSC-EV effects on SARS-CoV2-induced severe pneumonia and BPD. To date, there have been no large, randomized, and placebo-controlled clinical trials aimed at assessing the effect of MSC-EVs on lung diseases due to a limited understanding of the molecular mechanisms involved. As a cell-free alternative therapeutic agent, research into MSC-EV remains in its infancy and many questions need to have definitive answers. Among the most urgent questions are: 1) which linage or origin is the best to isolate the EVs? 2) which systemic (intravenous, intraperitoneal) or local (intratracheal, intrabronchial, intrapleural, intranasal) routes are suitable for various kinds of lung diseases? 3) Of single or multiple administration, which is superior? 4) Does MSC-EV therapy play a protagonist or adjuvant role in different lung diseases? 5) Which quali-quantitative compositions play key roles in the biological effects of MSC-EVs? 6) What are the gold-standard animal models for human lung diseases to confirm the MSC effects? For instance, in research into the effect of MSC-EVs on IPF, bleomycin-induced lung injury is mostly chosen to mimic the IPF microenvironment. This is regarded as the animal model which is most akin to the human pathophysiology of IPF, but the processes that drive IPF are complex, and bleomycin-induced lung injury is widely used in pulmonary fibrosis without considering its pathogenesis.

The other challenge of MSC-EV application in lung diseases for rapid translation from bench to bedside is the absence of standardized and consolidated criteria of EV production and separation. The differences in EV isolation and characterization retain a great deal of heterogeneous features that are at odds with the homogeneity required for the clinic. The members of four academic societies (ISCT, ISCT, ISBT, and SOCRATES) have identified the key defining physical and biological characteristics of MSC-sEVs in a position paper, but it did not mention how to predict the therapeutic potency of MSC-EVs within quantifiable and reproducible parameters [141]. Obtaining a deep understanding of MSC-sEV biology and developing an appropriate functional assay to test its therapeutic properties will facilitate the development of MSC-sEVs as an off-the-shelf alternative treatment for lung diseases.

What still remains a mystery is the appropriate therapeutic doses for each lung disease. Due to the lack of standard protocols of EV isolation and characterization, studies from different institutes are using various methods to quantify MSC-EVs. Protein concentration or particle amount of MSC-EVs are widely used to illustrate the dose of MSC-EVs in a large number of publications. The lack of standard protocols contributes to the doubtful and confusing results for clinicians if they plan to design clinical trials. Furthermore, the optimal dose for the phenotypes of lung diseases is also largely unknown. For example, ARDS subphenotypes, comprising hypo-inflammatory and hyper-inflammatory phenotypes, have been identified by Calfee *et al* [142], but which subphenotype is more suitable for MSC-EV treatment remains uncertain.

Finally, further investigations into how to scale up and develop the specific protocol of good manufacturing practice (GMP) for MSC-EV production are required as they are needed in large quantities based on the experimental respiratory animal model. Furthermore, it has been identified that the senescence of BM-MSCs, ADSCs or UC-MSCs may limit their use for isolating large-scale MSC-EVs, but embryonic stem cell-derived MSCs have shown the ability to produce large amounts of MSC-EVs with no changed in quantity and quality [143, 144]. Nevertheless, even though these publications provide some instructions for the biomanufacturing of MSC-EVs [145, 146], the field of large-scale EV biomanufacturing schemes remains unexplored.

## Future Perspectives

Respiratory diseases still threaten millions of people in all regions of the world and the strategies for their prevention and control are urgently required. More public attention and research funding should be given to respiratory diseases due to the increasing population and deteriorating environment. The findings concerning MSC-EV effects on pulmonary regeneration are consistent, and their potent capacities in immunomodulation are evident. The fact that MSC-EVs show less potential for immunogenicity and tumorigenicity contributes to the possibility of their clinical application in lung diseases, and the beneficial effects of their targeting immunoregulation and tissue repair result in the possibility of numerous biological advantages in the treatment of acute and chronic lung injury. Accordingly, substantial work in dissecting the exact molecular mechanisms of MSC-EV effects is required by further investigation. Otherwise, MSC-based cell-free alternative therapeutic regimens will remain imprecise and speculative.
